# The PI3K-Akt-mTOR pathway mediates renal pericyte-myofibroblast transition by enhancing glycolysis through HKII

**DOI:** 10.1186/s12967-023-04167-7

**Published:** 2023-05-13

**Authors:** Liangmei Chen, Xiaofan Li, Yiyao Deng, Jianwen Chen, Mengjie Huang, Fengge Zhu, Zhumei Gao, Lingling Wu, Quan Hong, Zhe Feng, Guangyan Cai, Xuefeng Sun, Xueyuan Bai, Xiangmei Chen

**Affiliations:** 1 Department of Nephrology, Chinese PLA General Hospital, Chinese PLA Institute of Nephrology, State Key Laboratory of Kidney Diseases, National Clinical Research Center of Kidney Diseases, Beijing Key Laboratory of Kidney Disease, Haidian District, Beijing, 100853 China; 2 Department of Nephrology, The First Affiliated Hospital of Jinan University, Jinan University, Tianhe District, Guangzhou, 510632 Guangdong China; 3 Department of Nephrology, Guizhou Provincial People’s Hospital, Guiyang, 550002 Guizhou China

**Keywords:** Pericyte-myofibroblast transition, TGF-β1, Glycolysis, PI3K-Akt-mTOR pathway, HKII

## Abstract

**Background:**

Pericyte-myofibroblast transition (PMT) has been confirmed to contribute to renal fibrosis in several kidney diseases, and transforming growth factor-β1 (TGF-β1) is a well-known cytokine that drives PMT. However, the underlying mechanism has not been fully established, and little is known about the associated metabolic changes.

**Methods:**

Bioinformatics analysis was used to identify transcriptomic changes during PMT. PDGFRβ + pericytes were isolated using MACS, and an in vitro model of PMT was induced by 5 ng/ml TGF-β1. Metabolites were analyzed by ultraperformance liquid chromatography (UPLC) and tandem mass spectrometry (MS). 2-Deoxyglucose (2-DG) was used to inhibit glycolysis via its actions on hexokinase (HK). The hexokinase II (HKII) plasmid was transfected into pericytes for HKII overexpression. LY294002 or rapamycin was used to inhibit the PI3K-Akt-mTOR pathway for mechanistic exploration.

**Results:**

An increase in carbon metabolism during PMT was detected through bioinformatics and metabolomics analysis. We first detected increased levels of glycolysis and HKII expression in pericytes after stimulation with TGF-β1 for 48 h, accompanied by increased expression of α-SMA, vimentin and desmin. Transdifferentiation was blunted when pericytes were pretreated with 2-DG, an inhibitor of glycolysis. The phosphorylation levels of PI3K, Akt and mTOR were elevated during PMT, and after inhibition of the PI3K-Akt-mTOR pathway with LY294002 or rapamycin, glycolysis in the TGF-β1-treated pericytes was decreased. Moreover, PMT and HKII transcription and activity were blunted, but the plasmid-mediated overexpression of HKII rescued PMT inhibition.

**Conclusions:**

The expression and activity of HKII as well as the level of glycolysis were increased during PMT. Moreover, the PI3K-Akt-mTOR pathway regulates PMT by increasing glycolysis through HKII regulation.

**Supplementary Information:**

The online version contains supplementary material available at 10.1186/s12967-023-04167-7.

## Background

Myofibroblasts secrete extracellular matrix and contribute to renal fibrosis [1–4], which is a hallmark of poor repair in acute kidney injury and progression to chronic kidney disease. In recent years, despite divergent opinions on the source of renal myofibroblasts [5–7], an increasing number of comprehensive genetic fate mapping studies have demonstrated that pericytes are the main progenitors of renal scar-forming myofibroblasts [2, 8–10]. Moreover, pericytes have been increasingly exploited in cell therapy to inhibit renal fibrosis and promote tissue regeneration [11–14].

Transforming growth factor β-1 (TGF-β1) is a pluripotent cytokine that drives organ fibrosis and regeneration [15, 16]. Following unilateral ureteral obstruction, injured epithelial cells primarily secrete TGF-β1, which can then trigger pericyte-myofibroblast transition (PMT) [16]. A previous study established an in vitro PMT model induced by TGF-β1 [16, 17].

Differentiation is a complicated process that involves dynamic epigenetic, transcriptional, and metabolic remodeling [18–20]. Metabolic remodeling, also known as metabolic reprogramming (MR), refers to the change in the primary production of ATP by cells from oxidative phosphorylation to aerobic glycolysis [21, 22]. Increasing research has revealed that metabolic reprogramming is no longer limited to changes in the balance of glycolysis and oxidative phosphorylation but extends to changes in the metabolism of various nutrients, such as fatty acids, amino acids and glutamine [23–25]. Studies have found a novel link between metabolism and epigenetic modulation in transdifferentiation [11, 20, 26], in which metabolites can regulate differentiation [18, 27]. PMT is an important pathological factor in the progression of renal fibrosis. However, changes in metabolic profiles and metabolic pathways in PMT remain poorly understood, and numerous questions regarding TGF-β1 biology and the pathophysiology of PMT remain unanswered.

In this study, we first report that glycolysis is increased during PMT and that the PI3K-Akt-mTOR pathway plays a role in this effect. Our data reveal that glycolysis and HKII expression levels were significantly elevated in PMT, and inhibiting glycolysis with an HKII competitive antagonist (2-DG) could reverse PMT. Moreover, when the PI3K-Akt-mTOR pathway was inhibited, PMT was blunted, accompanied by decreased HKII expression and activity. An HKII rescue experiment further clarified that the PI3K-Akt-mTOR pathway affects TGF-β1-induced PMT by regulating HKII.

## Methods

### Bioinformatics analysis

To identify the main transcriptomic changes that accompany the process of PMT, we downloaded the gene expression profiles of GSE50439 [28], whose raw data were extracted with the GPL1261 platform, (Mouse430_2) Affymetrix Mouse Genome 430 2.0 Array. In this database, Col1a1-eGFPL10a mice were subjected to sham or unilateral ureteral obstruction (UUO) surgery. From the mice subjected to the sham surgery, we extracted samples GSM1219324, GSM121932 and GSM1219326 as the control group, while from those subjected to UUO after 2 or 7 days, we extracted GSM1219330, GSM1219331, GSM1219332, GSM1219333, GSM1219334 and GSM1219335 as the PMT group. The preprocessing of the gene expression profile data, which included background correction, quantile normalization, median polish summarization, and log2 transformation, was performed by R and RStudio software. The limma R package was applied to identify differentially expressed genes (DEGs) by comparing expression values between pericyte samples and myofibroblast samples. The criteria for assessing DEGs were |log2-fold change (FC)|≥ 1 and *p* < 0.05 in the gene expression between pericytes and myofibroblasts and an adjusted* p* < 0.05. A volcano plot of DEGs was generated by the ggplot2 package in R. GO and Kyoto Encyclopedia of Genes and Genomes (KEGG) enrichment pathway analyses of the DEGs were performed with the Database for Annotation, Visualization and Integrated Discovery (DAVID) online tool.

### Metabolic profiling

Samples were collected and immediately stored at −80 °C according to previous literature [29]. Samples were extracted with 80% methanol aqueous solution and analyzed by ultraperformance liquid chromatography (UPLC) and tandem mass spectrometry (MS). Metabolite quantification was performed by using multiple reaction monitoring (MRM) mode in a triple-quadrupole mass spectrometer. Analyst 1.6.3 software was used to process the mass spectrometry data. Based on the local metabolic database, the metabolites of the samples were analyzed qualitatively and quantitatively by mass spectrometry.

### Primary pericyte isolation, culture and cell treatment

Pericytes were isolated from C57/BL6 WT mice (male, 6–8 weeks old, purchased from the Animal Center of Chinese PLA General Hospital) by separating the kidneys aseptically, then dicing and incubating them with Liberase (0.5 mg/ml, Roche Applied Science, USA) and DNase (100 U/ml, Roche Applied Science, USA) at 37 °C for 30 min in DMEM (Corning, USA). Digestion was inactivated with DMEM containing 10% FBS (Corning, USA). The suspension was filtered with a 40 μm cell strainer to remove glomeruli and multicellular debris. Pericytes were purified by isolating PDGFRβ + cells using MACS (Miltenyi Biotech, Germany) and cultured in DMEM-F12 (Gibco Life Technologies, USA) with 10% FBS, 1% penicillin/streptomycin (Gibco Life Technologies, USA) and 1% ITS (Invitrogen, USA) in collagen-type I-coated (Sigma‒Aldrich, USA) T25 flasks. P1 primary pericytes were used for experiments. Additional details on the above procedure can be found in the referenced literature [16, 30, 31]. Pericytes were pretreated with 5 mmol/L 2DG (Sigma‒Aldrich, USA), 50 μmol/L LY294002 (HY-10108, MCE, USA) or 100 nmol/L rapamycin (HY-10219, MCE, USA) to inhibit glycolysis [32] or the PI3K-Akt-mTOR pathway [33–35].

### Model of PMT in vitro

When the cell confluence reached approximately 80%, the culture medium was replaced with 1640 medium without glucose and serum and cultured for 12 h. After the cells were synchronized, they were divided into two groups: (1) the control group, in which pericytes were cultured with complete culture medium, and (2) the TGF-β1-treated group, in which pericytes were cultured with complete culture medium containing 5 ng/mL TGF-β1 (1218209, PeproTech) for 24 or 48 h [16]. The cell phenotype was verified by determining the expression levels of α-SMA, vimentin and desmin. According to our results, we considered that the in vitro PMT model was successfully established by 48 h of treatment with TGF-β1.

### Quantitative real-time polymerase chain reaction (PCR) analysis

Briefly, the cells were homogenized using TRIzol (Invitrogen, USA), and the total RNA was reverse-transcribed to cDNA by a ProtoScript II FirstStrand cDNA Synthesis Kit (Applied Biosystems, USA). Real-time PCR was carried out (Bio-Rad, USA) with FastStart Universal SYBR Green Master Mix (Roche, Germany). The primers are shown in Table S1. The relative mRNA level was determined with the 2^−ΔΔCt^ method normalized by 18S as described previously [36, 37].

### Western blotting

Cells were lysed in RIPA buffer with 1% PMSF on ice for 20 min, and the protein concentrations were evaluated with a BCA protein assay kit (Thermo, Rockford, IL, USA). The same quality proteins were separated in 10–15% polyacrylamide gel and then transferred to NC membranes by Trans-Blot Turbo (Bio-Rad, Hercules, CA, USA). After the membranes were blocked in 20% casein, they were incubated overnight with the following primary antibodies: GAPDH (1/50000, 60004–1, Proteintech), α-SMA (1/3000, ab7817, Abcam), vimentin (1/10000, 60330, Proteintech), desmin (1/1000, sc-23879, Santa Cruz Biotechnology), hexokinase II (HKII) (1/1000, ab209847, Abcam), PKM (1/5000, ab150377, Abcam), and LDH (1/5000, ab52488, Abcam). Quantification was performed by measuring the intensity of the gels with ImageJ (Rawak Software, Inc. Germany).

### Immunofluorescence staining

Immunofluorescence staining was described previously [38]. Cells cultured on coverslips were washed twice with cold PBS and fixed with 4% paraformaldehyde for 3 min at room temperature and 15 min at 4℃. Following three extensive washes with PBS, the slides were permeabilized with 0.2% Triton X-100 for 10 min, blocked with 5% BSA for 30 min at room temperature, and then incubated with the specific primary antibodies previously described. The slides were probed with Cy3‐conjugated secondary antibody (red) and FITC‐conjugated secondary antibody (green) at room temperature for 1 h. The slides were imaged by confocal fluorescence microscopy. Each experiment was repeated three times, and immunofluorescence images were captured with identical exposure settings. The signal intensity of immunofluorescence images was quantified with the same parameter settings using ImageJ software, and scores were expressed as the percentage of total area.

### Extracellular flux (XF) analysis

P1 pericytes were seeded in an XF 24-well cell culture microplate (Seahorse Bioscience, Copenhagen, Denmark) at a density of 2 × 10^4^ cells/well and washed in glucose-free XF base medium. A Seahorse XFe24 Extracellular Flux Analyzer was used to measure the extracellular acidification rate (ECAR) and the oxygen consumption rate (OCR) in the medium [39]. The ECAR was measured after serial injections with 100 mmol/L D-glucose, 10 μmol/L oligomycin, and 500 mmol/L 2-deoxyglucose. OCR was measured after serial injections with 15 μmol/L oligomycin, 20 μmol/L FCCP, and 5 μmol/L rotenone/antimycin A. Glycolysis was defined as the ECAR response to oligomycin after glucose injection, and the glycolytic reserve was calculated as ΔECAR _Oligomycin-Glucose_. The maximum OCR value stimulated by FCCP was defined as the maximal respiration, and the difference between the maximum respiration and the basal respiration was the spare respiratory capacity. Data were analyzed by the XF Glycolysis Stress Test Report Generator macro provided by Seahorse Biosciences.

### HK activity assays

We measured HK activity by using an HK activity assay kit (MAK091, Sigma, USA). In brief, glucose is converted to glucose-6-phosphate, which is oxidized by glucose-6-phosphate dehydrogenase to form NADH. The resulting NADH reduces a colorless probe, resulting in a colorimetric product (at λ = 450 nm). The HK activity was accurately measured by a microplate reader (Thermo Fisher, USA).

### Transformation of the HKII plasmid and transfection into pericytes

The pCMV6 plasmid (EX-Mm03044-M02, GeneCopoeia) containing the HKII cDNA sequence was transformed into TOP10 competent *E. coli* (CB104, Tiangen). After amplification, the plasmid was extracted with a Plasmid Mini Kit II according to the manufacturer’s protocol (D6945, Omega). When the cell confluence reached approximately 50%, the HKII plasmid was transfected into P1 pericytes with EndoFectin™ Max Transfection Reagent (EF003, GeneCopoeia) according to the instructions. The expression of HKII was measured by PCR.

### Statistical analyses

All Western blot and immunofluorescence images are representative of at least three independent experiments. RT‒qPCR assays were performed in quadruplicate. Data shown are the mean ± SD for three or more independent experiments. Student’s t test was used for two-group comparisons. P < 0.05 indicated statistical significance.

## Results

### Bioinformatics and metabolomics analysis revealed significant changes in the carbon metabolism pathway during PMT

To study the major changes that accompany PMT in transcriptomics, we extracted GSE50439 data from the GEO database. We analyzed gene expression in the bound fraction, which reflects pericyte/myofibroblast-specific RNA [28]. Sham-treated kidneys were used as the control group, and UUO-treated kidneys collected on day 2 or day 7 were used as the PMT group. By comparing the gene expression profiles of the two groups, we identified 1485 DEGs (Additional file 1: Fig. S1), which were enriched in 58 signaling pathways (data not shown). Among these pathways, 14 were related to metabolism (Additional file 1: Fig. S2). These results led us to further investigate the carbon metabolic changes during PMT. In the carbon metabolism pathway, we noticed that HKII levels changed significantly (data not shown).

**Fig. 1:**
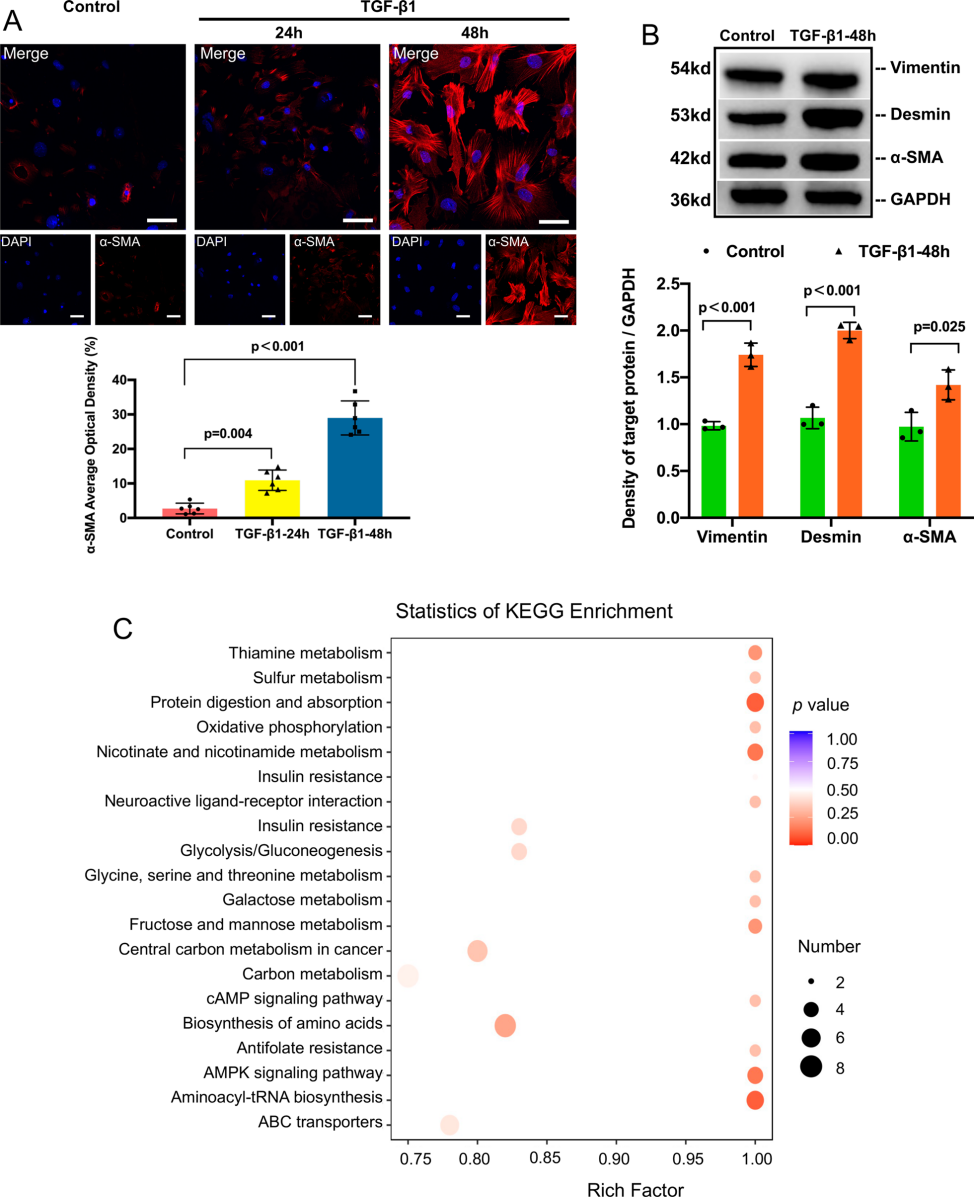
Carbon metabolism was changed in TGF-β1-induced PMT. **A** Immunofluorescence staining of α-SMA (red) and nuclei (blue) and quantitative data. Scale bar = 50 μm. **B** Representative Western blotting and quantitative data of vimentin, desmin and α-SMA expression after TGF-β1 stimulation. **C** Bubble chart of the pathways that were enriched in the 31 differentially expressed metabolites between pericytes and pericytes/myofibroblasts. Data are presented as the mean ± SD (n = 3–6)

Then, we established an in vitro model of PMT induced by TGF-β1. Primary pericytes were isolated from C57BL/6 mice using magnetic bead sorting. After the pericytes (passage 2) were stimulated with 5 ng/mL TGF-β1 for 24 h, the cells presented with a myofibroblast-like morphology (Additional file 1: Fig. S3), along with a prominently increased expression of α-SMA (Fig. 1A–B), consistent with previous reports [17]. After stimulation with TGF-β1 for 48 h, the increased expression of α-SMA, desmin and vimentin was more significant (Fig. 1A–B). Thus, we regarded pericytes treated with TGF-β1 for 48 h as myofibroblasts.

By using the PMT model established above, we conducted targeted metabolomic profiling of carbon metabolism in pericytes and TGF-β1-induced myofibroblasts. The results showed that 31 metabolites in carbon metabolism were increased in myofibroblasts (data not shown). Then, we performed KEGG analysis on the 31 metabolites and found that these metabolites were enriched in both glycolysis and oxidative phosphorylation (Fig. 1C).

### Glycolysis in carbon metabolism was prominently enhanced in TGF-β1-induced PMT

**Fig. 2:**
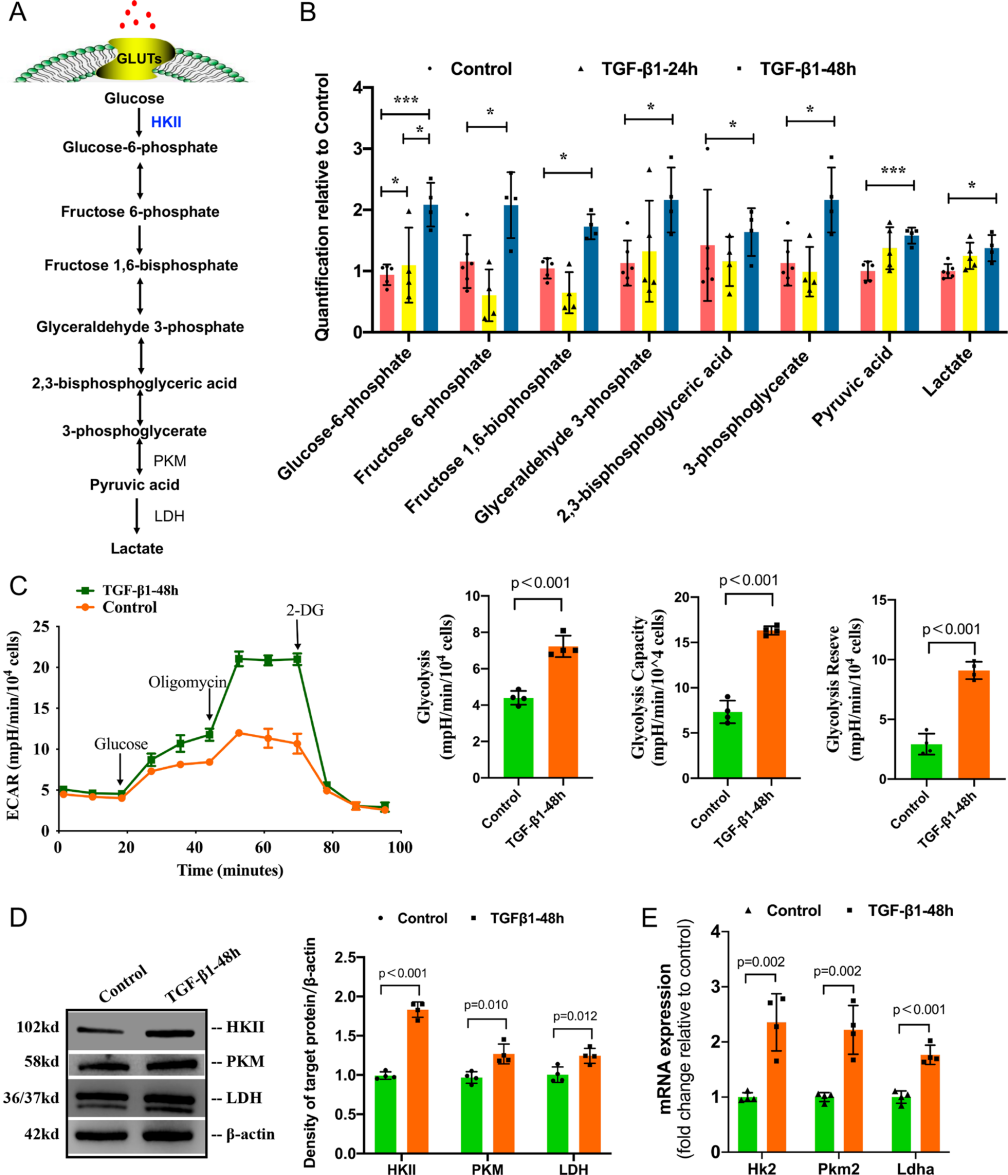
Glycolysis in carbon metabolism was prominently enhanced during TGF-β1-induced PMT. Pericytes stimulated with 5 ng/ml TGF-β1 for 48 h were recognized as myofibroblasts.** A** Schematic diagram of glycolysis; **B** Change in the levels of glycolysis metabolites during PMT. Control: inactive pericyte, TGF-β1-24 h: pericyte stimulated with 5 ng/ml TGF-β1 for 24 h, TGF-β1-48 h: pericyte stimulated with 5 ng/ml TGF-β1 for 48 h. **C** Extracellular acidification rate (ECAR) in pericytes and myofibroblasts, followed by sequential treatments with glucose, oligomycin A, and 2-deoxyglucose (2-DG). The level of glycolysis, glycolytic capacity and glycolytic reserve in the TGF-β1-48 h group were higher than those of the control group. **D**, **E** Representative Western blots and the gene expression of key glycolytic enzymes. Data are presented as the mean ± SD (n = 3–4)

A schematic illustration of glycolysis is shown in Fig. 2A. We first observed the accumulation of glycolytic metabolites, such as glucose-6-phosphate, fructose 6-phosphate, fructose 1,6-bisphosphate, glyceraldehyde 3-phosphate, 2,3-bisphosphoglyceric acid, 3-phosphoglycerate, pyruvic acid and lactate, during PMT (Fig. 2B).

To further define the metabolic profile, we examined the extracellular acidification rate (ECAR), which allows the quantification of glycolytic flux. The cells were first incubated in medium without glucose and pyruvate, and we found that the nonglycolytic acidification rate was unchanged during PMT (Fig. 2C). Nevertheless, the acidification rate increased further after injection of glucose and oligomycin in the TGF-β1-48h group, indicating a substantial improvement in glycolysis and glycolytic capacity. Additionally, the glycolytic reserve was improved in the TGF-β1-48h group compared to the control group. These results confirmed an increase in glycolysis during PMT.

We also detected the OCR as a measure of mitochondrial oxidative phosphorylation. As shown in Additional file 1: Fig. S4, compared with the control group, the TGF-β1-48h group had higher basal mitochondrial respiration, maximal respiration, and spare respiration capacity (p<0.05), but there was no significant difference in the ratio of the spare respiration capacity/basal respiration between the two groups (Additional file 1: Fig. S4E). Moreover, the ratio of glycolysis reserve/glycolysis was higher in the TGF-β1-48h group (Additional file 1: Fig. S4F). Therefore, glycolysis has a greater potential to supply energy during PMT.

As overall glycolysis increased, we also detected the uptake and consumption of glucose during PMT. The expression of glucose transporter 1 (p=0.006) and glucose transporter 4 (p=0.024) was significantly elevated in the TGF-β1-48h group relative to the control group (Additional file 1: Fig. S5). Furthermore, through the relative quantitative detection of glucose, we found that the glucose content in the TGF-β1-48h group was higher than that in the control group (p<0.001) (Additional file 1: Fig. S6A). In addition, the glucose concentration in the culture medium of the TGF-β1-48h group was significantly lower than that in the culture medium of the control group (p=0.013) (Additional file 1: Fig. S6B).

Having validated the increase in glucose uptake and consumption, we then measured changes in the expression of key glycolytic enzymes. Western blot analysis revealed increased expression levels of HKII, M2 pyruvate kinase (PKM) and lactate dehydrogenase (LDH) in the TGF-β1-48h group compared with the control group (Fig. 2D). TaqMan PCR analysis showed that the mRNA expression levels of HKII, Pkm2 and Ldhα were significantly elevated in the TGF-β1-48h group (Fig. 2E).

### Reducing glycolysis levels significantly inhibited TGF-β1-induced PMT

**Fig. 3:**
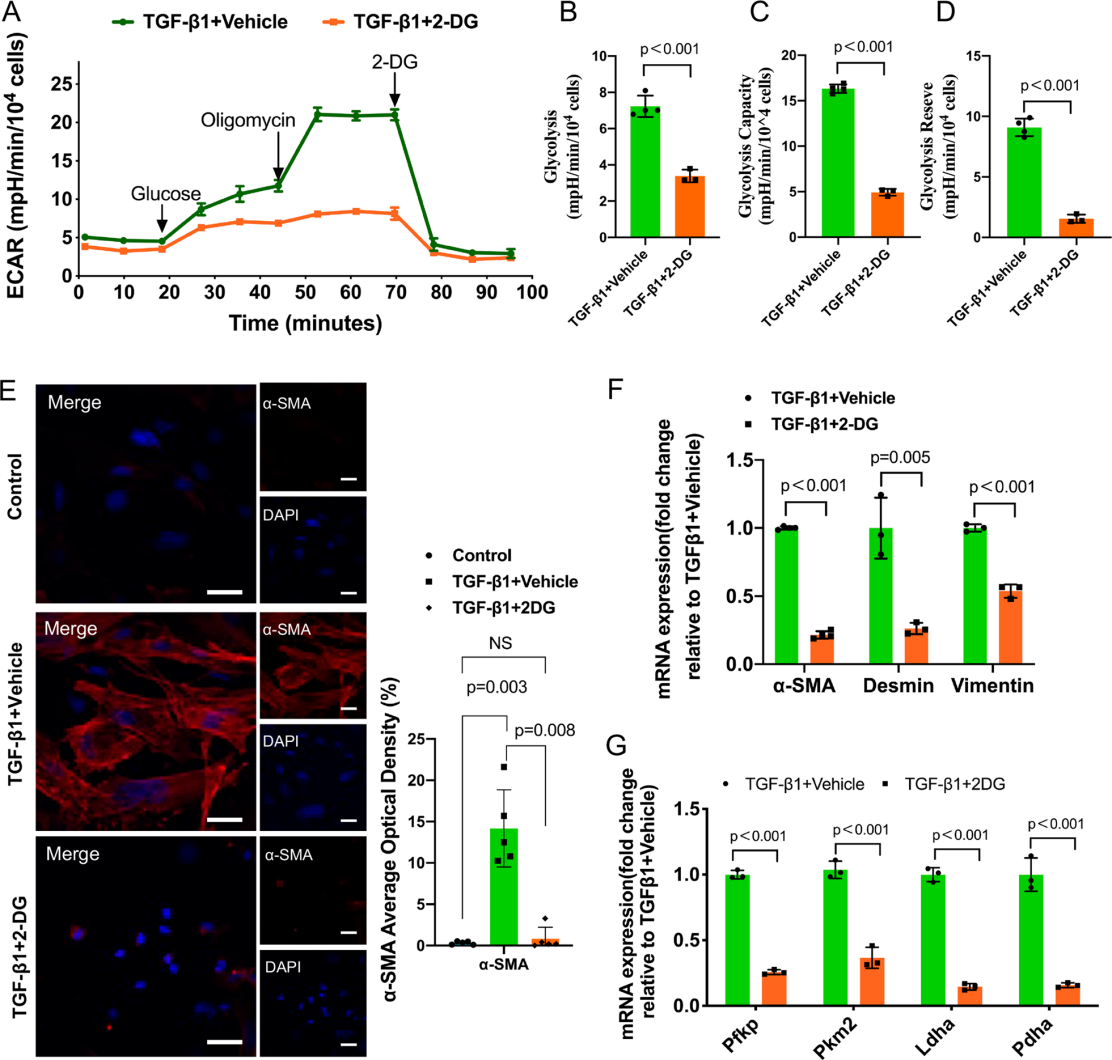
Reducing glycolysis levels significantly inhibited TGF-β1-induced PMT. 2-Deoxyglucose (2-DG) was used to reduce the level of glycolysis.** A** Effects of 2-DG on the ECAR in TGF-β1-induced PMT.** B**, **C** and** D** Statistical analyses of glycolysis, glycolytic capacity and glycolytic reserve in the ECAR. **E** Immunofluorescence staining of α-SMA (red) and nuclei (blue). Scale bar = 50 μm. **F** Gene expression of α-SMA, vimentin and desmin. **G** Gene expression of glycolytic enzymes downstream of HKII in the glycolytic pathway. Data are presented as the mean ± SD (n = 3–5)

The increase in glycolysis during PMT led us to investigate whether decreasing glycolysis can affect PMT. Thus, we pretreated pericytes with 2-DG, a glucose analog that inhibits glycolysis via its effect on HK, to answer this question. First, we applied the XFe24 Extracellular Flux Analyzer for real-time analysis of ECAR. As shown in Fig. 3A, compared with that of the TGF-β1 + vehicle group, the ECAR of the TGF-β1 + 2-DG group was significantly reduced. More importantly, the level of glycolysis, glycolytic capacity and glycolytic reserve were significantly decreased after 2-DG treatment (Fig. 3B-D). These results suggested that 2-DG significantly decreased the level of glycolysis.

Moreover, immunofluorescence showed that the expression of α-SMA was significantly reduced after 2-DG treatment (Fig. 3E). RT‒qPCR showed that the protein and mRNA levels of vimentin, desmin and α-SMA were decreased in the TGF-β1 + 2-DG group compared with the TGF-β1 + vehicle group (Fig. 3F). Since 2-DG acts on HK, we also analyzed the expression of other downstream glycolytic enzymes, such as PFKP, PKM2, LDHA and pyruvate dehydrogenase A (PDHA), and the results showed that the mRNA expression of these enzymes was reduced after the addition of 2-DG (Fig. 3G). Therefore, reducing the level of glycolysis can inhibit PMT as well as downstream glycolytic enzymes.

### Inhibiting the PI3K-Akt-mTOR pathway can reverse TGF-β1-induced PMT

**Fig. 4:**
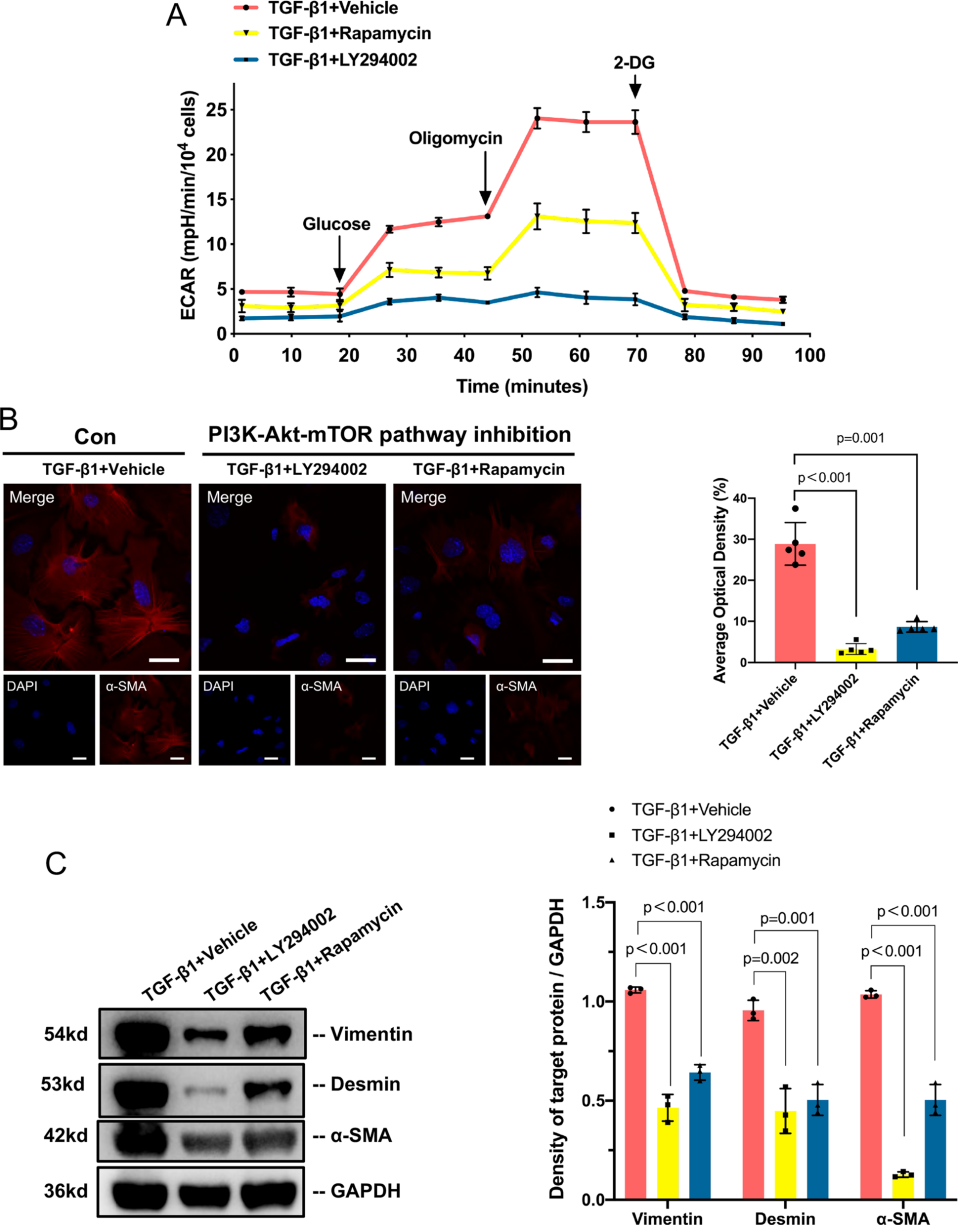
Inhibition of the PI3K-Akt-mTOR pathway reverses TGF-β1-induced PMT. **A** Effects of inhibition of the PI3K-Akt-mTOR pathway on the ECAR in TGF-β1-induced PMT. P1 pericytes were first treated with DMSO (^TGF−β1+ vehicle^), the PI3K inhibitor LY290042 (^TGF−β1+ LY290042^) or the mTOR inhibitor rapamycin (.^TGF−β1+rapamycin^) for 1 h, followed by the addition of TGF-β1 to a concentration of 5 ng/ml. **B** Immunofluorescence staining and quantitative detection of α-SMA (red) and nuclei (blue) before and after inhibiting the PI3K-Akt-mTOR pathway. Scale bar = 50 μm.** C** Representative Western blotting and quantitative data of α-SMA, vimentin and desmin. Data are presented as the mean ± SD (n = 3–5)

In the bioinformatics analysis, we noticed that the DEGs were enriched in the PI3K-Akt pathway during PMT. Then, we detected the phosphorylation levels of PI3K, Akt and mTOR and found that the PI3K-Akt-mTOR pathway was activated in PMT (Additional file 1: Fig. S7). Next, we treated the PMT model in vitro with LY294002 (a PI3K inhibitor) or rapamycin (an mTOR inhibitor) to specifically inhibit the PI3K-Akt-mTOR pathway. These treatments resulted in a decrease in the ECAR in the PMT model (Fig. 4A). The levels of glycolysis, glycolytic capacity and glycolytic reserve (all p<0.01) were significantly reduced (Additional file 1: Fig. S8).

Since lowering glycolysis can reduce PMT, we then evaluated pericyte transition after inhibition of the PI3K-Akt-mTOR pathway. The fluorescence intensity of α-SMA was notably reduced after treatment with LY294002 and rapamycin (Fig. 4B). The Western blotting results also showed that the expression levels of vimentin, desmin and α-SMA decreased in the TGF-β1 + LY294002 group and the TGF-β1 + rapamycin group (Fig. 4C).

### The PI3K-Akt-mTOR pathway influences TGF-β1-induced PMT by regulating HKII

**Fig. 5:**
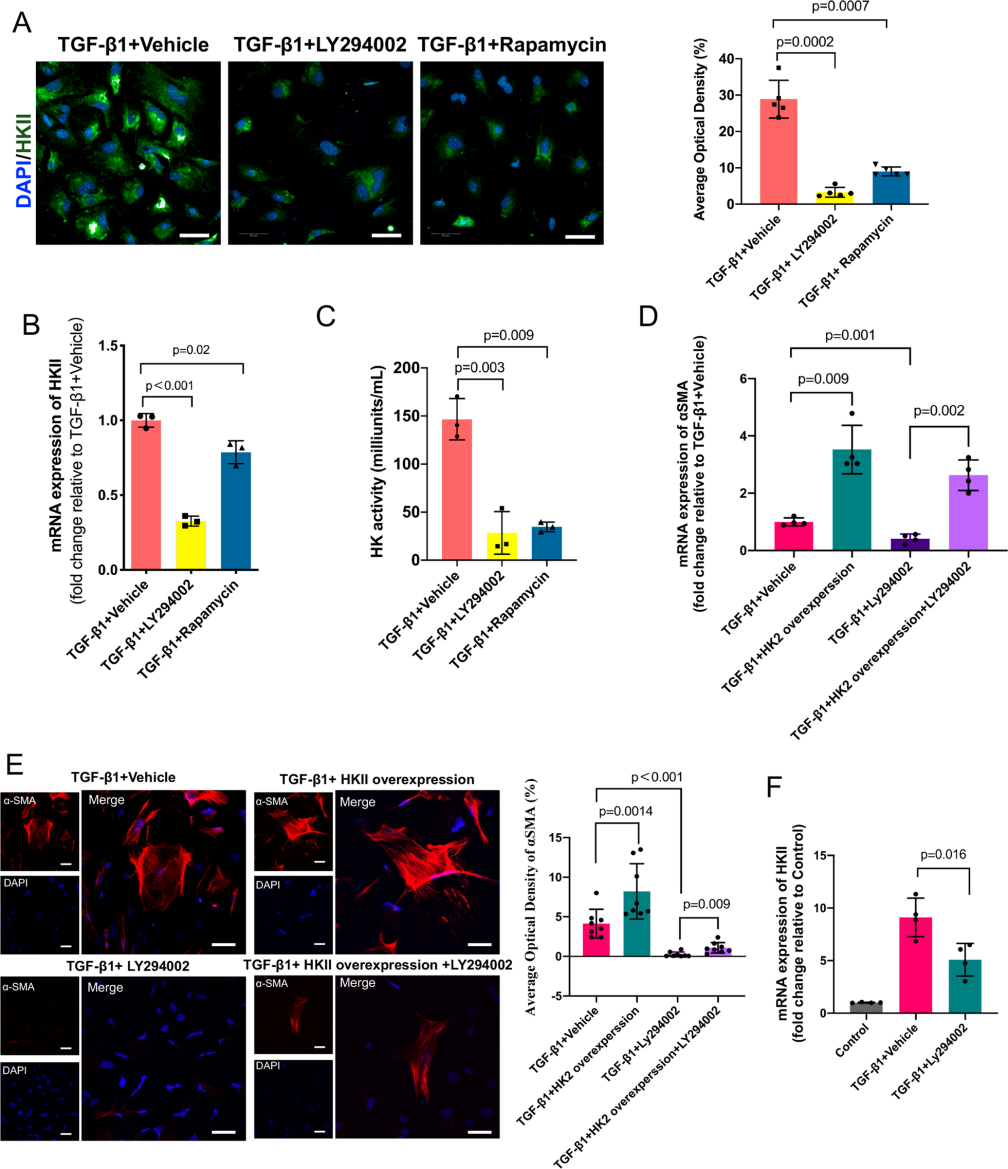
The PI3K-Akt-mTOR pathway influences TGF-β1-induced PMT by regulating HKII. **A** Immunofluorescence staining of HKII (green) and nuclei (blue) and quantitative data with or without inhibition of the PI3K-Akt-mTOR pathway. **B** Gene expression of HKII with or without inhibition of the PI3K-Akt-mTOR pathway. **C** HK activity with or without inhibition of the PI3K-Akt-mTOR pathway. **D**, **E** HKII rescue experiments showed that HKII overexpression rescued PMT induced by TGF-β1.** F** LY294002 decreased the mRNA expression of HKII. Scale bar = 50 μm. Data are presented as the mean ± SD (n = 3–8)

Thus far, the results demonstrated that inhibiting the PI3K-Akt-mTOR pathway reduced the level of glycolysis and suppressed PMT, but how the PI3K-Akt-mTOR pathway regulates PMT remains unclear. As we noticed a significant change in the level of HKII by bioinformatics analysis, consistent with a previous study, we hypothesized that HKII may play a key role in PMT. We found that the protein and mRNA levels of HKII were decreased in the PMT model after treatment with LY294002 or rapamycin (Fig. 5A–B). We then analyzed the activity of HK and found that it was also lower after treatment with LY294002 and rapamycin (Fig. 5C).

**Fig. 6:**
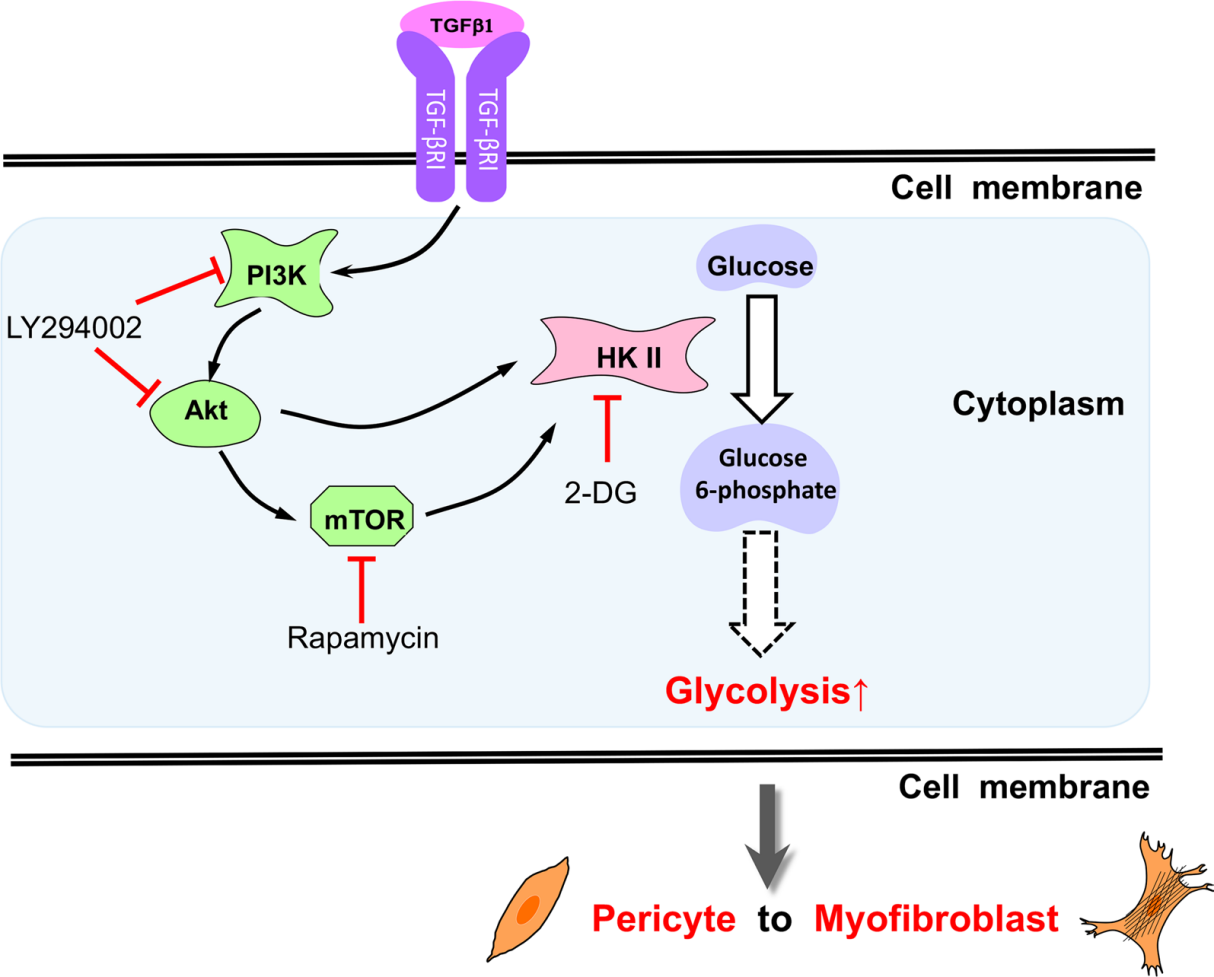
Schematic illustration showing that the PI3K-Akt-mTOR pathway regulates PMT by affecting HKII in glycolysis

Then, we performed an HKII rescue experiment. We successfully overexpressed HKII by plasmid transfection into pericytes (Additional file 1: Fig. S9), after which the pericytes were treated with TGF-β1 and LY294002. As shown in Fig. 5D–E, after HKII was overexpressed, PMT was significantly increased. Compared with PMT upon treatment with only LY294002, the overexpression of HKII also significantly increased PMT. Moreover, we detected lower mRNA expression levels of HKII in the PMT model after LY2940002 treatment. All these data suggest that the PI3K-Akt-mTOR pathway increases TGF-β1-induced PMT by enhancing the expression and activation of HKII (Fig. 6).

## Discussion

In this study, we compared the gene expression profiles of renal pericytes and pericyte-derived myofibroblasts through bioinformatics analysis. The results illustrated that cell metabolism changes significantly during PMT, involving carbon metabolism, HKII and the PI3K-Akt pathway. Then, we built an in vitro model of PMT to observe the specific changes in glycolysis. Moreover, we found that the PI3K-Akt-mTOR pathway may participate in the regulation of PMT by affecting HKII and glycolysis.

Renal fibrosis is the basic pathological feature of the progression of various renal diseases, and myofibroblasts are regarded as the predominant effector cells in fibrosis. However, the origins of myofibroblasts remain controversial, and possibilities include resident fibroblasts, pericytes, bone marrow-derived fibroblasts, tubular epithelial cells and endothelial cells [5, 7, 10, 40, 41]. In 2021, Kuppe C et al. published an article in Nature, and they identified distinct subpopulations of pericytes and fibroblasts as the main cellular sources of scar-forming myofibroblasts during human kidney fibrosis by using single-cell RNA sequencing and spatial transcriptomics in human samples and genetic fate tracing, time-course single-cell RNA sequencing and ATAC-seq experiments in mice [42]. Since an increasing number of in vitro and in vivo studies have confirmed that PMT is the main source of renal myofibroblasts in both humans and rodents [10, 42, 43], the role of PMT in renal diseases is self-evident.

The relationship between glycometabolism and renal disease has attracted the attention of many researchers. The mRNA and protein levels of key enzymes in glycolysis, as well as glycolysis levels, have been shown to be elevated in fibroblasts in a UUO mouse model [44], endothelial cells [45] and macrophages [46] in diabetic kidney disease (DKD). Inhibiting aerobic glycolysis can attenuate the progression of polycystic kidney disease, DKD, proliferative kidney disease, chronic kidney disease and kidney cancer [45, 47–50]. Our study revealed the changes in carbon metabolism in PMT, especially the role of the PI3K-Akt-mTOR pathway. We believe that our study may provide new ideas for preventing renal fibrosis and renal disease.

Metabolic studies on pericytes vary between pericyte subpopulations. Placental pericytes in the proliferative state rely heavily on glycolysis, which is responsible for producing approximately 85% of their ATP [51], while our results suggest that the level of ATP produced by mitochondria is 2–7 times that produced by glycolysis in primary renal pericytes at different cell densities (Additional file 1: Fig. S10D). The difference may be partly explained by the long-term hypoxia in malignant microenvironments and the abundant blood and oxygen supply in the kidney. Both GLUT1 and GLUT4 are detectable in renal pericytes, indicating that glucose uptake occurs through both insulin-dependent and insulin-independent pathways in pericytes [26], consistent with studies in murine brain pericytes [52]. Under TGF-β1 stimulation, the expression of GLUT1 and GLUT4 was increased, and the level of glucose in culture medium was lower in myofibroblasts (data not shown). These results illustrate that glucose uptake and consumption are higher in myofibroblasts than in pericytes. Cells in a quiescent state usually have low cellular metabolism, but when stimulated by growth factors, myofibroblasts increase ATP production to support cellular contractility [53]. Researchers have observed increased glycolysis and oxidative phosphorylation during the differentiation of neurons, podocytes and lung fibroblasts [53–55]. Moreover, metabolites such as the oxidized form of nicotinamide adenine dinucleotide (NAD^+^) can orchestrate transcription and differentiation [56]. Studies have found that glycolysis mainly provides most of the energy in the early stage of neuronal cell differentiation [54]. However, the levels of most metabolites begin to increase in the late stage of TGF-β1–induced PMT. N-cadherin, Notch3 and FoxO3A signaling not only participate in preserving cell‒cell interactions but also maintain a quiescent state in pericytes by limiting cellular metabolism, mainly glycolysis [11, 57, 58]. After blocking PFKFB3 with 3PO, glycolysis in pericytes is reduced, proliferation and migration are impaired, and attachment to epithelial cells by N-cadherin is enhanced [51]. After inhibiting the expression of PDK4 in human pulmonary pericytes, a similar phenomenon is observed, and the interaction between endothelial cells and pericytes is enhanced, thereby preventing vascular loss in pulmonary hypertension [59].

Renal pericytes, which have been neglected by researchers for years, are mesenchymal cells that surround endothelial cells in the capillary bed and postcapillary venules [60]. In recent studies, PMT has become a tenable target for preventing renal fibrosis [9, 61]. Pericytes activated by platelet-derived growth factor receptor signaling, TGF-β1 signaling or the Wnt pathway can detach from capillaries and transdifferentiate into myofibroblasts [16, 62]. TGF-β1 is a primary cytokine that stimulates PMT. In our study, pericytes cultured with TGF-β1 for 48 h strongly expressed α-SMA (a myofibroblast marker), while primary pericytes weakly expressed α-SMA.

TGF-β1 is a key mediator in the development of renal fibrosis, and our study demonstrated that TGF-β1 activated the PI3K-Akt-mTOR pathway and participated in pericyte differentiation by increasing glycolysis. Indeed, TGF-β regulates renal fibrosis via both canonical and noncanonical pathways [63]. TGF-β exerts biological effects by activating Smad2 and Smad3, which are negatively regulated by an inhibitory Smad7 [64]. In our bioinformatics analysis, even though the gene expression of most Smads (including Smad1-6) did not differ between pericytes and myofibroblasts derived from pericytes, we found that Smad7 is downregulated during renal fibrosis (data not shown), which is consistent with previous reports [65, 66]. The overexpression of Smad7 has been shown to be a therapeutic agent for renal fibrosis in various models of kidney diseases [66]. Moreover, Smads also interact with other signaling pathways, such as the mitogen-activated protein kinase (MAPK) and nuclear factor-κB (NF-κB) pathways [65], to positively or negatively regulate renal fibrosis. In our previous bioinformatics analysis, 27 DEGs were highly enriched in the MAPK signaling pathway, including MAPK9, MAPK11, MAPK13, MAPK14, MAPK8IP1 and MAPK8IP2 (data not shown). Some studies have demonstrated that the MAPK signaling pathway may participate in neural or cardiovascular pericyte differentiation [67–69], but whether the TGF-β1/MAPK pathway participates in renal pericyte metabolism and differentiation needs further study.

The results of bioinformatics analysis indicated that the PI3K-Akt-mTOR pathway may be involved in PMT; we verified that the PI3K-Akt-mTOR pathway participated in the regulation of PMT by regulating HKII in glycolysis. Some studies have indicated that Akt stimulates the phosphorylation of HKII and results in the protection of mitochondria against oxidant- or Ca^2+^-stimulated permeability transition pore opening [70]. In addition to playing an important role in cell proliferation, apoptosis and metabolism in malignant cells and neural stem cells [71–73], the PI3K-Akt-mTOR pathway is essential in cell pluripotency and differentiation [74]. It has been reported that when activated by insulin and insulin-like growth factor 1 (IGF1), the PI3K-Akt pathways maintain cell pluripotency by inhibiting the MEK/ERK signaling pathway [74].

In the present study, we first described metabolic reprogramming with enhanced glycolysis as a characteristic of PMT, including increased glucose consumption and an elevated level of glycolysis, and we demonstrated the vital role of the PI3K-Akt-mTOR pathway in PMT. Our study further suggests that the PI3K-Akt-mTOR pathway may affect glycolysis by mediating HKII. In addition, our work has implications not only for the fundamental understanding of metabolic reprogramming in PMT but also in prompting new ideas for inhibiting renal fibrosis. This research, however, is subject to several limitations. Although our research is based on previous transcriptome data analysis, it has not been verified in animal experiments, which will also be our future work. Meanwhile, the PMT model is induced by TGF-β1, and TGF-β1 is a multifunctional cytokine that is interconnected with multiple pathways. To explain how this complex network plays a role in PMT, more in-depth and systematic research is needed.

## Conclusion

The levels of glycolysis and HKII expression are increased during PMT. Reducing the level of glycolysis or inhibiting the PI3K-Akt-mTOR pathway inhibits PMT. The PI3K-Akt-mTOR pathway regulates PMT by increasing glycolysis through HKII regulation.

## Supplementary Information

**Additional file 1: Table S1.** Primer sequences used for RT‒PCR. **Figure S1.** Volcano map of DEGs between the control group and the PMT group, where the red and blue dots represent the upregulated genes (884) and downregulated genes (601) with respect to the control group. Transcriptomics data were obtained from GSE50439, and DEGs were screened with |log2-fold change (FC) |≥ 1 and *P* < 0.05. **Figure S2.** Bubble chart of metabolic-related pathways according to KEGG enrichment analysis results. **Figure S3.** Changes in morphology during PMT. **A** Pericyte without TGF-β1 stimulation; **B** Pericytes were stimulated with TGF-β1 for 24 h. **C** TUNEL staining results of pericyte with or without TGF-β1 treatment. Scale bar = 50 μm. **Figure S4.** Changes in oxidative phosphorylation levels during PMT. **A** Mitochondrial stress test curve of Seahorse XF cells. **B** Basal respiration level. **C** Maximum respiration. **D** Spare respiratory capacity. **E** The ratio of the spare respiratory capacity to the basal respiratory level. **F** The ratio of glycolysis reserve to glycolysis. n = 4, the results are expressed as the mean ± standard deviation. **Figure S5.** qRT‒PCR results showing that glucose transporter expression was increased in myofibroblasts. **Figure S6.** The level of intracellular glucose **A** and Extracellular glucose levels in the control group and TGF-β1-48 h group** B**. **Figure S7.** The PI3K-Akt-mTOR pathway is activated in PMT. **A** Representative Western blotting of p-PI3K, PI3K, p-Akt, Akt, p-mTOR and mTOR. **B**, **C** and **D** Phosphorylation levels of PI3K, Akt and mTOR. **Figure S8.** Inhibition of the PI3K-Akt-mTOR pathway reduced glycolysis **A** glycolytic capacity,** B** and glycolytic reserve, **C** in the model of TGF-β1-induced PMT. Data are presented as the mean ± SD (*n* = 3). **Figure S9.** The HKII plasmid was successfully transfected into pericytes. Data are presented as the mean ± SD (*n* = 3). **Figure S10.** Assay to detect the real-time change in the ATP level in pericytes with a Seahorse analyzer. **A** Kinetic curve showing the OCR.** B** Kinetic curve showing the ECAR. **C** The ATP production rate and **D** ATP rate index in pericytes at different seeding densities. **Figure S11.** Full blots for all Western blot experiments.

## Data Availability

The data used to support the findings of this study are included within the article. The data and materials in the current study are available from the corresponding author on reasonable request.

## Abbreviations

PMT: Pericyte-myofibroblast transition
UUO: Unilateral ureteral obstruction
HKII: Hexokinase II
PI3K: Phosphatidyl-inositol 3-kinase
mTOR: Mammalian target of rapamycin
GLUT: Glucose transporter
TGF‑β1: Transforming growth factor β1
MR: Metabolic reprogramming
PKM2: Pyruvate kinase isozymes M2
