# Supplementary material for: The PI3K-Akt-mTOR pathway mediates renal pericyte-myofibroblast transition by enhancing glycolysis through HKII
Source: J Transl Med. 2023 May 13;21:323. doi: 10.1186/s12967-023-04167-7 (PMC10182641; doi:10.1186/s12967-023-04167-7)
Supplement: Supplementary file 1 — Additional file 1: Table S1. Primer sequences used for RT‒PCR. Figure S1. Volcano map of DEGs between the control group and the PMT group, where the red and blue dots represent the upregulated genes (884) and downregulated genes (601) with respect to the control group. Transcriptomics data were obtained from GSE50439, and DEGs were screened with |log2-fold change (FC) |≥ 1 and P < 0.05. Figure S2. Bubble chart of metabolic-related pathways according to KEGG enrichment analysis results. Figure S3. Changes in morphology during PMT. A Pericyte without TGF-β1 stimulation; B Pericytes were stimulated with TGF-β1 for 24 h. C TUNEL staining results of pericyte with or without TGF-β1 treatment. Scale bar = 50 μm. Figure S4. Changes in oxidative phosphorylation levels during PMT. A Mitochondrial stress test curve of Seahorse XF cells. B Basal respiration level. C Maximum respiration. D Spare respiratory capacity. E The ratio of the spare respiratory capacity to the basal respiratory level. F The ratio of glycolysis reserve to glycolysis. n = 4, the results are expressed as the mean ± standard deviation. Figure S5. qRT‒PCR results showing that glucose transporter expression was increased in myofibroblasts. Figure S6. The level of intracellular glucose A and Extracellular glucose levels in the control group and TGF-β1-48 h group B. Figure S7. The PI3K-Akt-mTOR pathway is activated in PMT. A Representative Western blotting of p-PI3K, PI3K, p-Akt, Akt, p-mTOR and mTOR. B, C and D Phosphorylation levels of PI3K, Akt and mTOR. Figure S8. Inhibition of the PI3K-Akt-mTOR pathway reduced glycolysis A glycolytic capacity, B and glycolytic reserve, C in the model of TGF-β1-induced PMT. Data are presented as the mean ± SD (n = 3). Figure S9. The HKII plasmid was successfully transfected into pericytes. Data are presented as the mean ± SD (n = 3). Figure S10. Assay to detect the real-time change in the ATP level in pericytes with a Seahorse analyzer. A Kinetic curve showing the OCR. B Ki [file 12967_2023_4167_MOESM1_ESM.docx]

**Additional Tables and Figures For**

**The PI3K-Akt-mTOR pathway mediates renal pericyte-myofibroblast transition** **by enhancing glycolysis through** **HK****II**

Liangmei Chen^1,2^^†^, Xiaofan Li^1†^, Yiyao Deng^3^, Jianwen Chen^1^, Mengjie Huang^1^, Fengge Zhu^1^, Zhumei Gao^1^, Lingling Wu^1^, Quan Hong^1^, Zhe Feng^1^, Guangyan Cai^1^, Xuefeng Sun^1^, Xueyuan Bai^1*^, Xiangmei Chen^1*^

**Table S1****. Primer sequences used for RT‒PCR**

| Gene | Forward Primer (5′ → 3′) | Reverse Primer (5′ → 3′) |  |
| --- | --- | --- | --- |
| α-Sma | CCCAGACATCAGGGAGTAATGG | TCTATCGGATACTTCAGCGTCA |  |
| Hk2 | ATGATCGCCTGCTTATTCACG | CGCCTAGAAATCTCCAGAAGGG |  |
| Glut1 | GCAGTTCGGCTATAACACTGG | GCGGTGGTTCCATGTTTGATTG |  |
| Glut4 | ACACTGGTCCTAGCTGTATTCT | CCAGCCACGTTGCATTGTA |  |
| Pkm2 | CGCCTGGACATTGACTCTG | GAAATTCAGCCGAGCCACATT |  |
| Ldha | CAAAGACTACTGTGTAACTGCGA | TGGACTGTACTTGACAATGTTGG |  |
| Pfkp | CGCCTATCCGAAGTACCTGGA | CCCCGTGTAGATTCCCATGC |  |
| 18S | GTAACCCGTTGAACCCCATT | CCATCCAATCGGTAGTAGCG |  |

**Additional Fig. 1** Volcano map of DEGs between the control group and the PMT group, where the red and blue dots represent the upregulated genes (884) and downregulated genes (601) with respect to the control group. Transcriptomics data were obtained from GSE50439, and DEGs were screened with |log2-fold change (FC)| ≥1 and *P*<0.05.


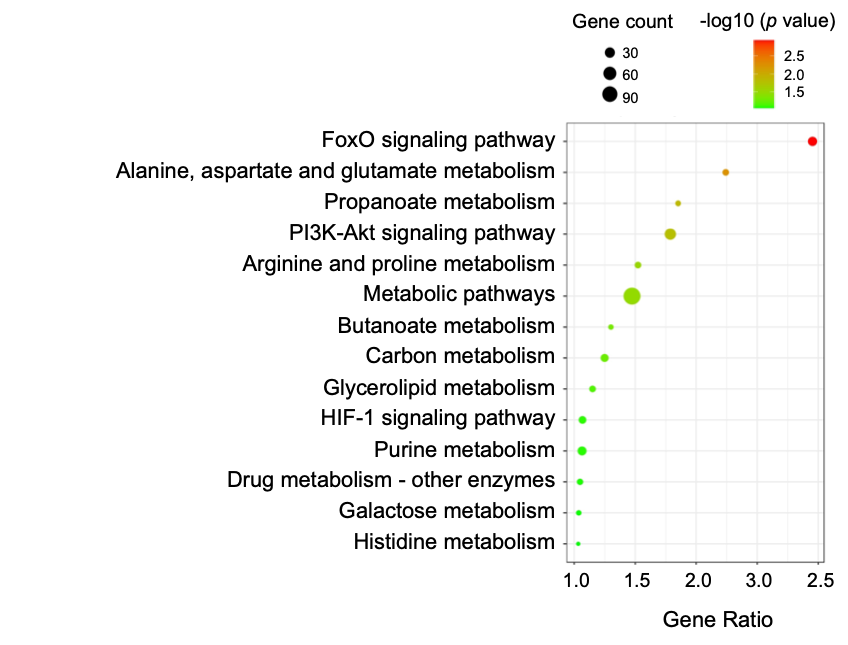


**Additional Fig. 2** Bubble chart of metabolic-related pathways according to KEGG enrichment analysis results.

**Additional Fig. 3** Changes in morphology during PMT. **A** Pericyte without TGF-β1 stimulation; **B** Pericytes were stimulated with TGF-β1 for 24 hours. **C** TUNEL staining results of pericyte with or without TGF-β1 treatment. Scale bar=50μm.

**Additional Fig. 4** Changes in oxidative phosphorylation levels during PMT. **A** Mitochondrial stress test curve of Seahorse XF cells. **B** Basal respiration level. **C** Maximum respiration. **D** Spare respiratory capacity. **E** The ratio of the spare respiratory capacity to the basal respiratory level. **F** The ratio of glycolysis reserve to glycolysis. n=4, the results are expressed as the mean ± standard deviation.


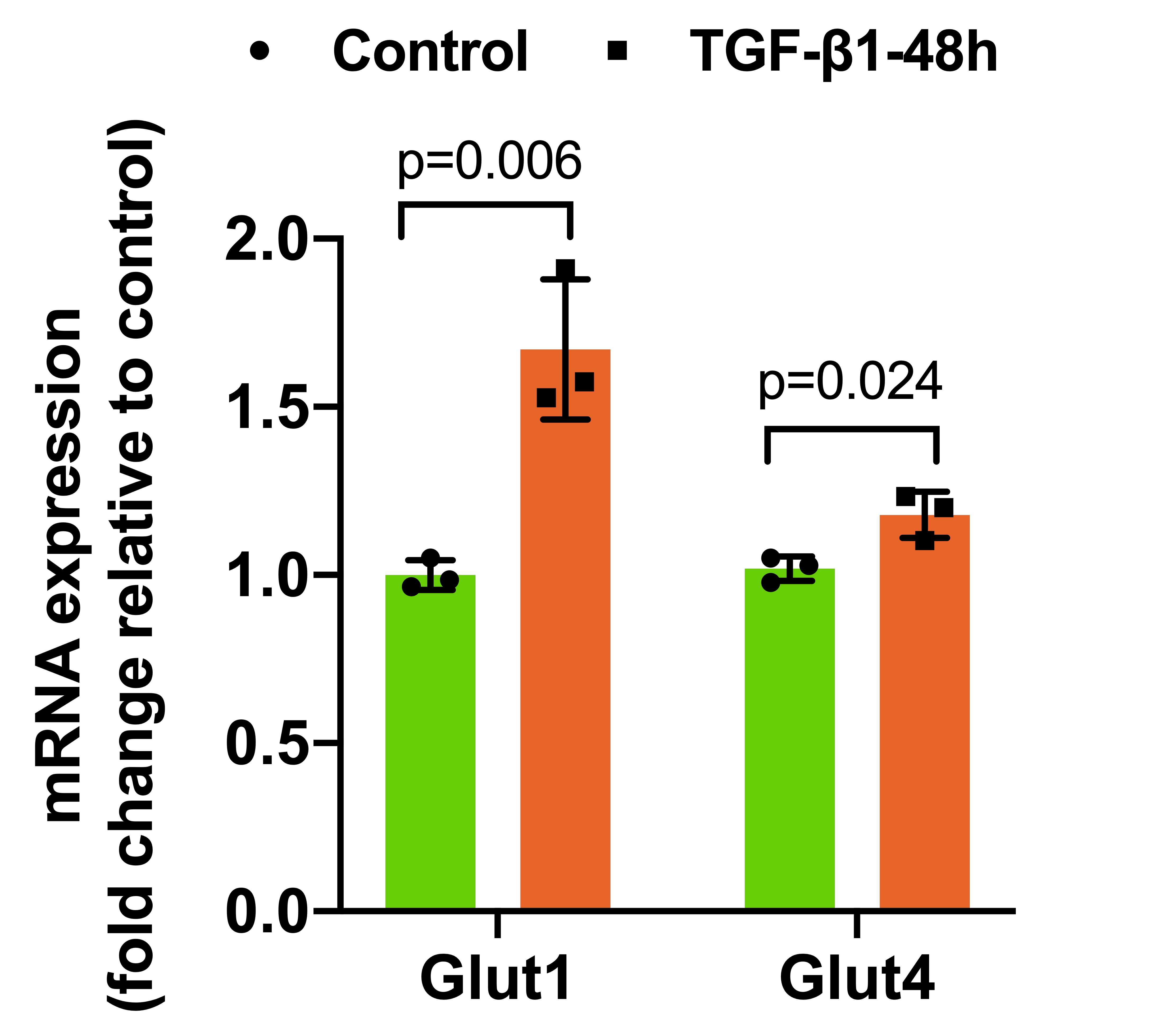


**Additional Fig. 5** qRT‒PCR results showing that glucose transporter expression was increased in myofibroblasts.

**Additional Fig. 6** The level of intracellular glucose **A** and Extracellular glucose levels in the control group and TGF-β1-48h group **B**.

**Additional Fig. 7** The PI3K-Akt-mTOR pathway is activated in PMT. **A** Representative Western blotting of p-PI3K, PI3K, p-Akt, Akt, p-mTOR and mTOR. **B**, **C** and **D** Phosphorylation levels of PI3K, Akt and mTOR.

**Additional Fig. 8** Inhibition of the PI3K-Akt-mTOR pathway reduced glycolysis **A** glycolytic capacity, **B** and glycolytic reserve, **C** in the model of TGF-β1-induced PMT. Data are presented as the mean ± SD (*n*=3-5).


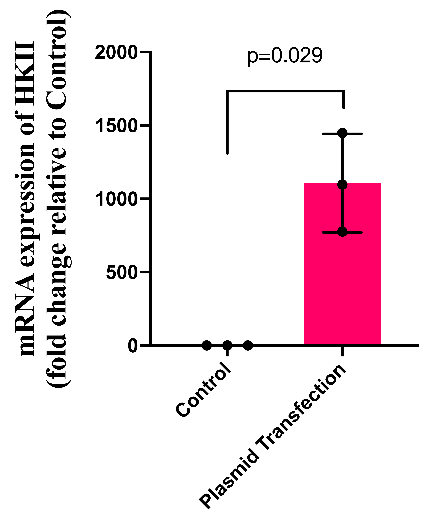


**Additional Fig. 9** The HKII plasmid was successfully transfected into pericytes. Data are presented as the mean ± SD (*n*=3).

**Additional Fig. 10** Assay to detect the real-time change in the ATP level in pericytes with a Seahorse analyzer. **A** Kinetic curve showing the OCR. **B** Kinetic curve showing the ECAR. **C** The ATP production rate and **D** ATP rate index in pericytes at different seeding densities.


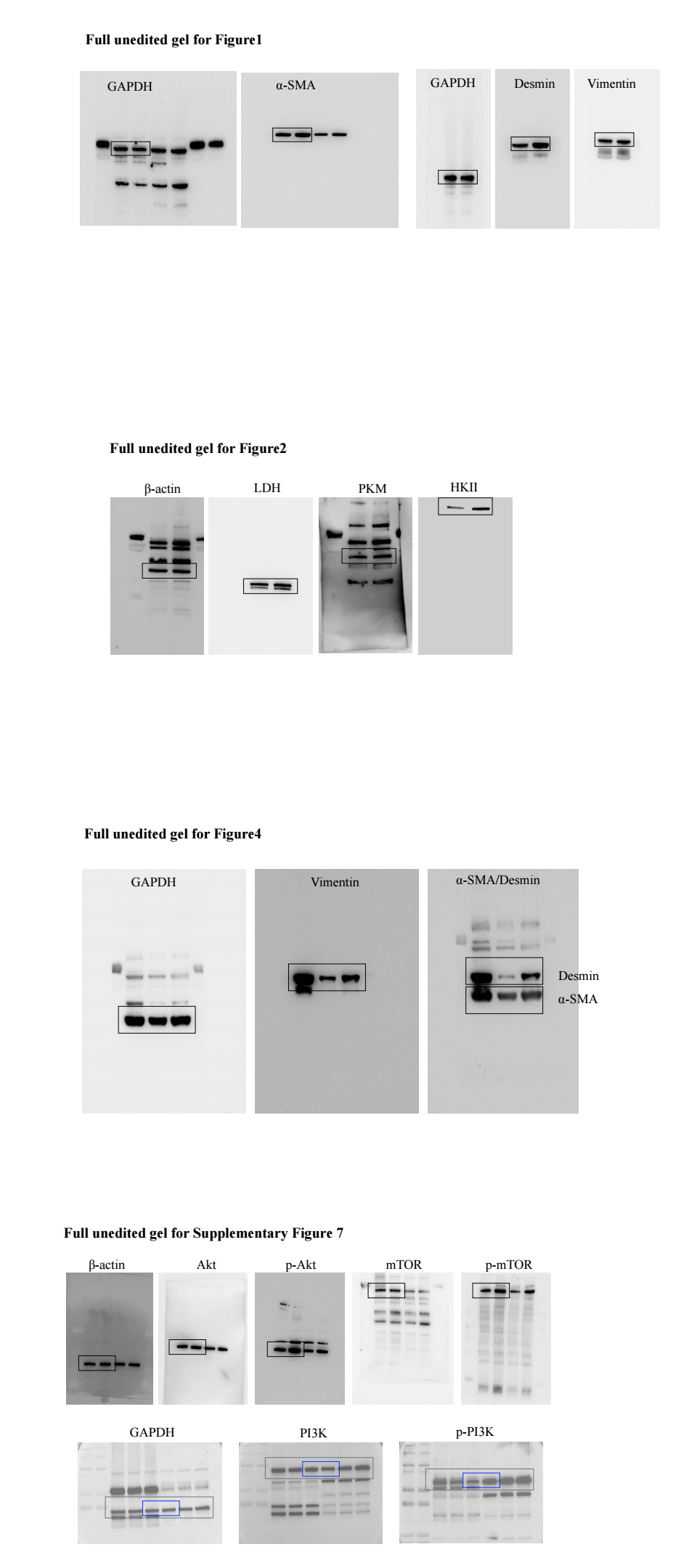


**Additional Figure 11.** Full blots for all Western blot experiments.
